# *N*′-[(2-Hydr­oxy-1-naphth­yl)methyl­idene]-2-nitro­benzohydrazide

**DOI:** 10.1107/S160053681001490X

**Published:** 2010-04-28

**Authors:** Yu-Mei Hao

**Affiliations:** a Department of Chemistry, Baicheng Normal University, Baicheng 137000, People’s Republic of China

## Abstract

In the title Schiff base compound, C_18_H_13_N_3_O_4_, prepared by the reaction of 2-hydr­oxy-1-naphthaldehyde with 2-nitro­benzohydrazide, the dihedral angle between the benzene ring and naphthyl ring system is 23.0 (2)°. There is an intra­molecular O—H⋯N hydrogen bond involving the naphthalene hydr­oxy substituent and a hydrazide N atom. In the crystal structure, symmetry-related mol­ecules are linked through inter­molecular N—H⋯O hydrogen bonds, forming chains propagating in [101].

## Related literature

For the pharmaceutical and medicinal activity of Schiff bases, see: Dao *et al.* (2000 ▶); Sriram *et al.* (2006 ▶); Karthikeyan *et al.* (2006 ▶). For the coordination chemistry of Schiff bases, see: Ali *et al.* (2008 ▶); Kargar *et al.* (2009 ▶); Yeap *et al.* (2009 ▶). For the crystal structures of Schiff base compounds, see: Fun *et al.* (2009 ▶); Nadeem *et al.* (2009 ▶); Eltayeb *et al.* (2008 ▶). For Schiff base compounds reported by the author, see: Hao (2009*a* ▶,*b* ▶,*c* ▶,*d* ▶). For reference structural data, see: Allen *et al.* (1987 ▶).
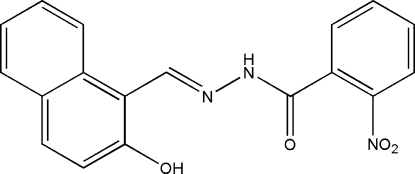


## Experimental

### 

#### Crystal data

C_18_H_13_N_3_O_4_
*M* *_r_* = 335.31
Monoclinic, 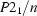

*a* = 7.4473 (6) Å
*b* = 29.068 (2) Å
*c* = 7.8504 (6) Å
β = 113.963 (4)°
*V* = 1553.0 (2) Å^3^
*Z* = 4
Mo *K*α radiation
μ = 0.10 mm^−1^
*T* = 298 K
0.30 × 0.28 × 0.27 mm

#### Data collection

Bruker SMART CCD area detector diffractometer
Absorption correction: multi-scan (*SADABS*; Sheldrick, 1996 ▶) *T*_min_ = 0.970, *T*_max_ = 0.973
8499 measured reflections
2972 independent reflections
1856 reflections with *I* > 2σ(*I*)
*R*_int_ = 0.038

#### Refinement

*R*[*F* ^2^ > 2σ(*F* ^2^)] = 0.044
*wR*(*F* ^2^) = 0.111
*S* = 1.05
2972 reflections
230 parameters
1 restraint
H atoms treated by a mixture of independent and constrained refinement
Δρ_max_ = 0.16 e Å^−3^
Δρ_min_ = −0.22 e Å^−3^

### 

Data collection: *SMART* (Bruker, 2007 ▶); cell refinement: *SAINT* (Bruker, 2007 ▶); data reduction: *SAINT*; program(s) used to solve structure: *SHELXS97* (Sheldrick, 2008 ▶); program(s) used to refine structure: *SHELXL97* (Sheldrick, 2008 ▶); molecular graphics: *SHELXTL* (Sheldrick, 2008 ▶); software used to prepare material for publication: *SHELXL97*.

## Supplementary Material

Crystal structure: contains datablocks global, I. DOI: 10.1107/S160053681001490X/su2174sup1.cif

Structure factors: contains datablocks I. DOI: 10.1107/S160053681001490X/su2174Isup2.hkl

Additional supplementary materials:  crystallographic information; 3D view; checkCIF report

## Figures and Tables

**Table 1:** Hydrogen-bond geometry (Å, °)

*D*—H⋯*A*	*D*—H	H⋯*A*	*D*⋯*A*	*D*—H⋯*A*
O1—H1⋯N1	0.82	1.87	2.5881 (18)	146
N2—H2⋯O2^i^	0.90 (1)	1.94 (1)	2.8133 (19)	164 (2)

Symmetry code: (i) 
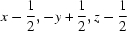
.

**Fig. 1.**
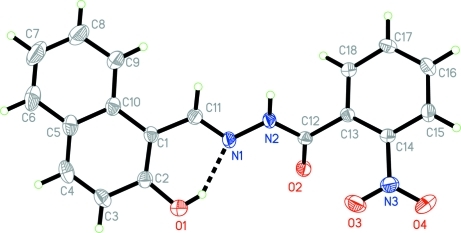
The molecular structure of the title compound with 30% probability ellipsoids. The intramolecular O-H···N hydrogenbond is shown as a dashed line.

**Fig. 2.**
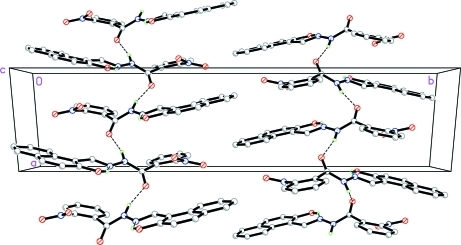
Molecular packing of the title compound, viewed along the c-axis, with the N-H···O hydrogen bonds drawn as dashed lines (see Table 1 for details).

## supplementary crystallographic information

### Comment

Schiff base compounds are a class of important materials used as pharmaceuticals and in various medicinal fields of interest (Dao *et al.*, 2000; Sriram *et al.*, 2006; Karthikeyan *et al.*, 2006). Schiff bases have also been used as versatile ligands in coordination chemistry (Ali *et al.*, 2008; Kargar *et al.*, 2009; Yeap *et al.*, 2009). Recently, the crystal structures of a large number of new Schiff base compounds have been reported (Fun *et al.*, 2009; Nadeem *et al.*, 2009; Eltayeb *et al.*, 2008). As a continuation of our work on such compounds (Hao, 2009a,b,c,d) we report herein on the crystal structure of a new title Schiff base compound, prepared by the reaction of 2-hydroxy-1-naphthyaldehyde with 2-nitrobenzohydrazide.

The molecular structure of the title compound is illustrated in Fig. 1. In the molecule there is an intramolecular O—H···N hydrogen bond, involving the naphthalene hydroxyl substituent and the hydrazide N-atom (Fig.1 and Table 1). The molecule is twisted with the dihedral angle between the benzene and the naphthyl ring mean planes being 23.0 (2)°. All the bond lengths are within normal values (Allen *et al.*, 1987).

In the crystal structure, symmetry related molecules are linked through intermolecular N—H···O hydrogen bonds, forming chains propagating in [101] (see Table 1 and Fig. 2).

### Experimental

2-Hydroxy-1-naphthyaldehyde (0.1 mmol, 17.2 mg) and 2-nitrobenzohydrazide (0.1 mmol, 18.1 mg) in 30 ml of methanol were refluxed for 30 min to give a clear colourless solution. Colourless block-shaped single crystals of the title compound were formed by slow evaporation of the solvent over several days at room temperature.

### Refinement

Hydrogen atom H2 was located in a difference Fourier map and refined isotropically, with the N—H distance restrained to 0.90 (1)Å, and *U*_iso_(H) restrained to 0.08 Å^2^. The other H-atoms were included in calculated positions and treated as riding atoms: d(C—H) = 0.93 Å, d(O—H) = 0.82 Å with *U*_iso_(H) = 1.2*U*_eq_(C) and 1.5*U*_eq_(O).

### Crystal data

**Table:**

C_18_H_13_N_3_O_4_	*F*(000) = 696
*M**_r_* = 335.31	*D*_x_ = 1.434 Mg m^−^^3^
Monoclinic, *P*2_1_/*n*	Mo *K*α radiation, λ = 0.71073 Å
Hall symbol: -P 2yn	Cell parameters from 1415 reflections
*a* = 7.4473 (6) Å	θ = 2.6–24.5°
*b* = 29.068 (2) Å	µ = 0.10 mm^−^^1^
*c* = 7.8504 (6) Å	*T* = 298 K
β = 113.963 (4)°	Block, colourless
*V* = 1553.0 (2) Å^3^	0.30 × 0.28 × 0.27 mm
*Z* = 4	

### Data collection

**Table:**

Bruker SMART CCD area detector diffractometer	2972 independent reflections
Radiation source: fine-focus sealed tube	1856 reflections with *I* > 2σ(*I*)
graphite	*R*_int_ = 0.038
ω scans	θ_max_ = 25.9°, θ_min_ = 2.8°
Absorption correction: multi-scan (*SADABS*; Sheldrick, 1996)	*h* = −9→9
*T*_min_ = 0.970, *T*_max_ = 0.973	*k* = −35→30
8499 measured reflections	*l* = −9→9

### Refinement

**Table:**

Refinement on *F*^2^	Primary atom site location: structure-invariant direct methods
Least-squares matrix: full	Secondary atom site location: difference Fourier map
*R*[*F*^2^ > 2σ(*F*^2^)] = 0.044	Hydrogen site location: inferred from neighbouring sites
*wR*(*F*^2^) = 0.111	H atoms treated by a mixture of independent and constrained refinement
*S* = 1.05	*w* = 1/[σ^2^(*F*_o_^2^) + (0.0464*P*)^2^ + 0.0232*P*] where *P* = (*F*_o_^2^ + 2*F*_c_^2^)/3
2972 reflections	(Δ/σ)_max_ < 0.001
230 parameters	Δρ_max_ = 0.16 e Å^−^^3^
1 restraint	Δρ_min_ = −0.22 e Å^−^^3^

### Special details

**Table:**

Geometry. All esds (except the esd in the dihedral angle between two l.s. planes) are estimated using the full covariance matrix. The cell esds are taken into account individually in the estimation of esds in distances, angles and torsion angles; correlations between esds in cell parameters are only used when they are defined by crystal symmetry. An approximate (isotropic) treatment of cell esds is used for estimating esds involving l.s. planes.
Refinement. Refinement of F^2^ against ALL reflections. The weighted R-factor wR and goodness of fit S are based on F^2^, conventional R-factors R are based on F, with F set to zero for negative F^2^. The threshold expression of F^2^ > 2sigma(F^2^) is used only for calculating R-factors(gt) etc. and is not relevant to the choice of reflections for refinement. R-factors based on F^2^ are statistically about twice as large as those based on F, and R- factors based on ALL data will be even larger.

### Fractional atomic coordinates and isotropic or equivalent isotropic displacement parameters (Å^2^)

**Table:**

	*x*	*y*	*z*	*U*_iso_*/*U*_eq_	
N1	0.9372 (2)	0.21679 (5)	0.1587 (2)	0.0397 (4)	
N2	0.8992 (2)	0.24623 (5)	0.0095 (2)	0.0391 (4)	
N3	0.8947 (3)	0.38594 (6)	0.0264 (2)	0.0484 (5)	
O1	0.9712 (2)	0.20162 (5)	0.49534 (18)	0.0565 (4)	
H1	0.9752	0.2167	0.4087	0.085*	
O2	1.1317 (2)	0.29693 (4)	0.18479 (17)	0.0468 (4)	
O3	0.8562 (2)	0.36371 (5)	0.1378 (2)	0.0621 (5)	
O4	0.9318 (3)	0.42719 (5)	0.0425 (2)	0.0876 (6)	
C1	0.8835 (3)	0.14285 (6)	0.2610 (3)	0.0380 (5)	
C2	0.9240 (3)	0.15757 (6)	0.4402 (3)	0.0411 (5)	
C3	0.9145 (3)	0.12727 (7)	0.5760 (3)	0.0517 (6)	
H3	0.9385	0.1380	0.6949	0.062*	
C4	0.8703 (3)	0.08250 (8)	0.5329 (3)	0.0570 (6)	
H4	0.8643	0.0628	0.6236	0.068*	
C5	0.8329 (3)	0.06477 (7)	0.3541 (3)	0.0506 (6)	
C6	0.7861 (4)	0.01817 (8)	0.3104 (4)	0.0742 (8)	
H6	0.7802	−0.0016	0.4012	0.089*	
C7	0.7492 (5)	0.00153 (8)	0.1374 (4)	0.0952 (10)	
H7	0.7182	−0.0294	0.1101	0.114*	
C8	0.7582 (4)	0.03098 (8)	0.0022 (4)	0.0897 (9)	
H8	0.7343	0.0195	−0.1156	0.108*	
C9	0.8014 (4)	0.07638 (7)	0.0383 (3)	0.0661 (7)	
H9	0.8057	0.0954	−0.0553	0.079*	
C10	0.8398 (3)	0.09508 (6)	0.2155 (3)	0.0439 (5)	
C11	0.8723 (3)	0.17566 (6)	0.1180 (3)	0.0402 (5)	
H11	0.8167	0.1666	−0.0063	0.048*	
C12	0.9971 (3)	0.28614 (6)	0.0366 (2)	0.0348 (4)	
C13	0.9339 (3)	0.31554 (6)	−0.1353 (2)	0.0331 (4)	
C14	0.8952 (3)	0.36234 (6)	−0.1387 (2)	0.0352 (5)	
C15	0.8493 (3)	0.38854 (7)	−0.2967 (3)	0.0479 (5)	
H15	0.8260	0.4199	−0.2947	0.057*	
C16	0.8383 (3)	0.36763 (7)	−0.4579 (3)	0.0528 (6)	
H16	0.8061	0.3849	−0.5660	0.063*	
C17	0.8747 (3)	0.32149 (7)	−0.4602 (3)	0.0487 (6)	
H17	0.8671	0.3076	−0.5696	0.058*	
C18	0.9226 (3)	0.29568 (6)	−0.3002 (2)	0.0406 (5)	
H18	0.9478	0.2644	−0.3029	0.049*	
H2	0.808 (3)	0.2378 (7)	−0.1025 (18)	0.080*	

### Atomic displacement parameters (Å^2^)

**Table:**

	*U*^11^	*U*^22^	*U*^33^	*U*^12^	*U*^13^	*U*^23^
N1	0.0456 (10)	0.0343 (9)	0.0344 (9)	−0.0023 (8)	0.0112 (8)	0.0062 (7)
N2	0.0450 (10)	0.0332 (9)	0.0301 (9)	−0.0065 (8)	0.0061 (8)	0.0051 (7)
N3	0.0534 (12)	0.0479 (11)	0.0422 (11)	0.0055 (9)	0.0176 (9)	−0.0051 (9)
O1	0.0807 (12)	0.0454 (9)	0.0459 (9)	−0.0007 (8)	0.0283 (9)	−0.0018 (7)
O2	0.0517 (9)	0.0414 (8)	0.0320 (7)	−0.0067 (7)	0.0014 (7)	0.0027 (6)
O3	0.0759 (12)	0.0751 (11)	0.0434 (9)	0.0037 (9)	0.0325 (9)	0.0006 (8)
O4	0.1449 (18)	0.0426 (10)	0.0877 (14)	−0.0041 (10)	0.0601 (13)	−0.0203 (9)
C1	0.0375 (12)	0.0358 (11)	0.0377 (11)	0.0007 (9)	0.0123 (9)	0.0057 (9)
C2	0.0415 (12)	0.0365 (11)	0.0443 (12)	0.0048 (9)	0.0165 (10)	0.0045 (9)
C3	0.0574 (15)	0.0562 (14)	0.0440 (13)	0.0084 (11)	0.0231 (11)	0.0134 (11)
C4	0.0537 (15)	0.0567 (15)	0.0612 (16)	0.0096 (11)	0.0241 (13)	0.0288 (12)
C5	0.0436 (13)	0.0421 (12)	0.0598 (15)	0.0019 (10)	0.0145 (11)	0.0140 (11)
C6	0.0713 (18)	0.0451 (14)	0.088 (2)	−0.0054 (13)	0.0132 (15)	0.0221 (13)
C7	0.121 (3)	0.0380 (14)	0.097 (2)	−0.0154 (15)	0.013 (2)	−0.0023 (15)
C8	0.131 (3)	0.0440 (15)	0.0764 (18)	−0.0076 (16)	0.0243 (18)	−0.0090 (14)
C9	0.091 (2)	0.0417 (13)	0.0582 (16)	−0.0089 (12)	0.0226 (14)	−0.0033 (11)
C10	0.0407 (12)	0.0372 (11)	0.0481 (12)	0.0010 (9)	0.0120 (10)	0.0063 (10)
C11	0.0405 (12)	0.0379 (11)	0.0376 (11)	−0.0001 (9)	0.0112 (9)	0.0011 (9)
C12	0.0373 (11)	0.0350 (11)	0.0291 (10)	0.0008 (9)	0.0104 (9)	−0.0013 (8)
C13	0.0311 (10)	0.0350 (10)	0.0300 (10)	−0.0032 (8)	0.0092 (8)	0.0003 (8)
C14	0.0366 (11)	0.0366 (11)	0.0303 (10)	−0.0017 (9)	0.0115 (9)	−0.0032 (8)
C15	0.0555 (14)	0.0377 (11)	0.0472 (13)	0.0027 (10)	0.0174 (11)	0.0089 (10)
C16	0.0670 (16)	0.0523 (14)	0.0358 (12)	−0.0011 (11)	0.0176 (11)	0.0114 (10)
C17	0.0616 (15)	0.0537 (13)	0.0313 (12)	−0.0040 (11)	0.0193 (11)	−0.0034 (10)
C18	0.0470 (13)	0.0376 (11)	0.0350 (11)	−0.0022 (9)	0.0145 (10)	−0.0023 (9)

### Geometric parameters (Å, °)

**Table:**

N1—C11	1.281 (2)	C6—C7	1.360 (3)
N1—N2	1.3838 (19)	C6—H6	0.9300
N2—C12	1.341 (2)	C7—C8	1.386 (4)
N2—H2	0.899 (9)	C7—H7	0.9300
N3—O3	1.212 (2)	C8—C9	1.360 (3)
N3—O4	1.225 (2)	C8—H8	0.9300
N3—C14	1.468 (2)	C9—C10	1.411 (3)
O1—C2	1.352 (2)	C9—H9	0.9300
O1—H1	0.8200	C11—H11	0.9300
O2—C12	1.229 (2)	C12—C13	1.502 (2)
C1—C2	1.381 (2)	C13—C18	1.388 (2)
C1—C10	1.438 (2)	C13—C14	1.389 (2)
C1—C11	1.449 (2)	C14—C15	1.375 (2)
C2—C3	1.407 (3)	C15—C16	1.376 (3)
C3—C4	1.351 (3)	C15—H15	0.9300
C3—H3	0.9300	C16—C17	1.370 (3)
C4—C5	1.413 (3)	C16—H16	0.9300
C4—H4	0.9300	C17—C18	1.380 (3)
C5—C6	1.406 (3)	C17—H17	0.9300
C5—C10	1.417 (3)	C18—H18	0.9300
			
C11—N1—N2	116.07 (15)	C7—C8—H8	119.3
C12—N2—N1	119.34 (15)	C8—C9—C10	121.0 (2)
C12—N2—H2	122.4 (14)	C8—C9—H9	119.5
N1—N2—H2	118.3 (14)	C10—C9—H9	119.5
O3—N3—O4	123.87 (18)	C9—C10—C5	117.49 (19)
O3—N3—C14	118.30 (17)	C9—C10—C1	123.50 (18)
O4—N3—C14	117.83 (18)	C5—C10—C1	119.01 (18)
C2—O1—H1	109.5	N1—C11—C1	121.60 (17)
C2—C1—C10	119.08 (17)	N1—C11—H11	119.2
C2—C1—C11	120.32 (17)	C1—C11—H11	119.2
C10—C1—C11	120.47 (17)	O2—C12—N2	123.57 (16)
O1—C2—C1	122.73 (17)	O2—C12—C13	122.88 (16)
O1—C2—C3	115.90 (17)	N2—C12—C13	113.46 (16)
C1—C2—C3	121.36 (18)	C18—C13—C14	117.16 (16)
C4—C3—C2	119.7 (2)	C18—C13—C12	118.64 (16)
C4—C3—H3	120.2	C14—C13—C12	124.12 (16)
C2—C3—H3	120.2	C15—C14—C13	122.20 (17)
C3—C4—C5	122.0 (2)	C15—C14—N3	116.68 (17)
C3—C4—H4	119.0	C13—C14—N3	121.09 (16)
C5—C4—H4	119.0	C14—C15—C16	119.03 (19)
C6—C5—C4	121.5 (2)	C14—C15—H15	120.5
C6—C5—C10	119.6 (2)	C16—C15—H15	120.5
C4—C5—C10	118.81 (19)	C17—C16—C15	120.40 (19)
C7—C6—C5	121.0 (2)	C17—C16—H16	119.8
C7—C6—H6	119.5	C15—C16—H16	119.8
C5—C6—H6	119.5	C16—C17—C18	120.01 (19)
C6—C7—C8	119.5 (2)	C16—C17—H17	120.0
C6—C7—H7	120.3	C18—C17—H17	120.0
C8—C7—H7	120.3	C17—C18—C13	121.19 (18)
C9—C8—C7	121.4 (3)	C17—C18—H18	119.4
C9—C8—H8	119.3	C13—C18—H18	119.4

### Hydrogen-bond geometry (Å, °)

**Table:**

*D*—H···*A*	*D*—H	H···*A*	*D*···*A*	*D*—H···*A*
O1—H1···N1	0.82	1.87	2.5881 (18)	146
N2—H2···O2^i^	0.90 (1)	1.94 (1)	2.8133 (19)	164 (2)

Symmetry codes: (i) *x*−1/2, −*y*+1/2, *z*−1/2.
